# Enquête d’opinion des praticiens ivoiriens sur la pratique de la rééducation périnéale dans le post-partum

**DOI:** 10.11604/pamj.2020.37.367.23426

**Published:** 2020-12-22

**Authors:** Edele Kacou Aka, Mohamed Fanny, Abdoul Koffi, N´guessan Olou, Apollinaire Horo, Mamourou Kone

**Affiliations:** 1 Université Félix Houphouët Boigny (FHB), Abidjan, Côte d´Ivoire,; 2 Service de gynécologie Obstétrique, Centre Hospitalier Universitaire de Yopougon, Yopougon, Côte d´Ivoire

**Keywords:** Troubles du plancher pelvien, médecine physique et réadaptation, connaissances, Afrique de l’Ouest, Pelvic floor disorders, physical medicine and rehabilitation, knowledge, West Africa

## Abstract

**Introduction:**

cette étude visait à rapporter l´expérience des prestataires de soins dans les maternités d´Abidjan en matière de rééducation périnéale en évaluant leur niveau de connaissance et leurs attitudes pratiques.

**Méthodes:**

une enquête a été menée auprès des obstétriciens et sages-femmes exerçant dans une maternité d´Abidjan depuis au moins deux ans. Les informations ont été recueillies sur un questionnaire à questions directes, ouvertes et semi-ouvertes.

**Résultats:**

cent vingt praticiens ont été sélectionnés dont 66 sages-femmes et 54 obstétriciens. Un total de 79,6% des obstétriciens et 37,9% des sages-femmes officiaient dans un centre hospitalier universitaire. L´indice de connaissance des sages-femmes était bon ou moyen dans 25% (intervalle interquartile [IIQ] 16,8-48,3). Tandis que les obstétriciens avaient un niveau de connaissances bon ou moyen dans 65% (IIQ 41,1-48,3). La majorité des prestataires convoquaient les femmes dans le post partum, mais seulement 7 soignants sur dix contrôlaient le périnée et deux obstétriciens sur dix proposaient une rééducation périnéale.

**Conclusion:**

le faible niveau de connaissances des sages-femmes et obstétriciens ivoiriens sur la rééducation périnéale du post-partum et le manque de pratique suggèrent la nécessité d´une mise à niveau perpétuelle de ceux-ci à travers des formations continues.

## Abstract (en)

**Introduction:**

the purpose of this study was to report health care providers’ experience of perineal rehabilitation in the Maternity Hospital of Abidjan by assessing their level of knowledge and their practical attitudes.

**Methods:**

we performed a survey among obstetricians and midwives working in a Maternity Hospital in Abidjan over a period of at least two years. The information were collected using a questionnaire including direct, open and semi-open questions.

**Results:**

the study included 120 practitioners, 66 midwives and 54 obstetricians. Seventy-nine point six percent of obstetricians and 37.9% of midwives worked in a university hospital. Midwifery knowledge was good or average in 25% of cases (interquartile range [IIQ] 16.8-48.3); 65% of obstetricians had a good or medium level of knowledge (IIQ 41.1 -48.3). The majority of providers summoned women in the postpartum period, but only 7 out of 10 caregivers performed perineal assessment and 2 out of 10 obstetricians suggested the need for rehabilitation.

**Conclusion:**

the low level of knowledge of Ivorian midwives and obstetricians regarding postpartum pelvic floor rehabilitation and the lack of practice suggest the need for ongoing training.

## Introduction

Les femmes qui recherchent un traitement pour un prolapsus des organes pelviens à un stade avancé ont une image corporelle et une qualité de vie globale réduites [1]. Quel que soit le mode d'accouchement, une augmentation de la distensibilité du muscle du releveur persiste après l'accouchement. Cette distensibilité accrue du plancher pelvien peut jouer un rôle dans le développement d'un dysfonctionnement du plancher pelvien plus tard dans la vie [2]. Parmi les TPP, une grande proportion de patientes présente une incontinence urinaire (IU) qui en représente une forme nosologique. En effet, la prévalence de l'IU peut atteindre 75,2% pendant la grossesse [3] et 37,9% dans la période puerpérale voire 12 ans après un accouchement [4]. Face à l´IU légère ou modérée, plusieurs auteurs estiment que la rééducation périnéale par un entrainement du plancher pelvien (EPP) est un traitement [3, 5]. L´EPP contribue également à la reprise de la vie sexuelle. Cependant, l'efficacité de cet entrainement est directement liée à l'adhésion des patientes à cette méthode. La mauvaise observance peut réduire considérablement les taux de succès [4-7]. L´implication des prestataires de soins de santé potentialise cette efficacité en favorisant l´adhésion et l´observance de celles-ci [8-11]. Peu de données existent en Afrique et en Côte d´Ivoire sur la rééducation périnéale. Le but de notre étude est donc de déterminer le degré d´implication des praticiens de santé dans l´entrainement du plancher pelvien dans le post partum.

## Méthodes

**Caractéristiques de l´étude:** nous avons effectué une enquête transversale à visée descriptive auprès des prestataires de soins (Gynécologues-obstétriciens et Sages-femmes) exerçant dans les maternités du district d´Abidjan, ayant au moins deux années d´expérience professionnelle et membre de la Société de Gynécologie-Obstétrique de Côte d´Ivoire (SOGOCI).

**Méthode d´investigation:** un questionnaire pré-test avec des questions ouvertes sur la connaissance des troubles du plancher pelvien et sa prise en charge par la rééducation périnéale a été distribué à des gynécologues-obstétriciens du CHU de Yopougon. Ce choix s´est porté sur ce centre abritant en son sein, le centre national de médecine physique et de réadaptation de Côte d´Ivoire. A partir de ce pré-test, les réponses recueillies nous ont permis de mettre en place un questionnaire standardisé avec une proposition de grille d´analyse du niveau de connaissances et les attitudes pratiques. Cette grille d´analyse est différente selon que l´on soit sage-femme ou obstétriciens spécialistes.

**Collecte des données:** l´enquête a été réalisée sur 3 mois du 1^er^ mars 2019 au 31 mai 2019. Un questionnaire standardisé anonyme avec 21 items; questions directes, ouvertes et semi-ouvertes sur la connaissance des troubles du plancher pelvien et sa prise en charge par la rééducation périnéale a été distribué aux praticiens. Une relance hebdomadaire par mail était systématique afin de maximiser le nombre de répondants.

**Variables étudiées:** outre les caractéristiques professionnelles, les autres paramètres étudiés étaient le niveau de connaissances (la présence de fuites urinaires au cours de la grossesse, les fuites urinaires comme motif de consultation, l´existence d´une rééducation fonctionnelle du post partum, les complications abdominales du post partum et les différentes méthodes de rééducation périnéale du post partum), les attitudes et pratiques (la recherche de l´incontinence urinaire comme signe fonctionnel pendant la grossesse, l´éducation les patientes sur l´incontinence urinaire, la proposition la rééducation périnéale pendant la grossesse, la convocation les patientes dans le post partum, les motifs de convocation dans le post partum, l´observance des rendez-vous de consultation et les méthodes proposées).

**Critère de jugement:** l´indice de connaissance qui est un objectif quantitatif a été restitué en 4 niveaux: (Mauvais, Insuffisant, Moyen ou Bon). Cette grille d´analyse fonction du nombre de bonnes réponses est selon la qualité des cibles. Ainsi, pour les obstétriciens (- de 50% de bonnes réponses = Mauvais; - de 65% de bonnes réponses = Insuffisant; - de 85% de bonnes réponses = Moyen; + de 85% de bonnes réponses = Bon) et pour les sages-femmes (- de 25% de bonnes réponses = Mauvais, - de 50% de bonnes réponses = Insuffisant, - de 70% de bonnes réponses = Moyen, et + de 70% de bonnes réponses = Bon).

**Saisie et analyse des données:** les données ont étés enregistrées, analysées et les résultats obtenus grâce aux logiciels épi info7 et Stata 14. Des statistiques descriptives étaient utilisées pour présenter les caractéristiques professionnelles. Les données catégorielles sont présentées sous forme de fréquence et de pourcentage. L´indice de connaissance était la somme des réponses données pour chaque item concernées par le dit indice de façon binaire avec 1 pour la bonne réponse et 0 lorsqu’elle n´était pas correcte.

**Considérations éthiques:** les informations ont été recueillies dans la stricte confidentialité à l´aide d´un questionnaire anonyme et les résultats ont été tenus secrets. Nous avons pris la peine d´informer le personnel de santé quant à l´intérêt des données recueillies et aussi avons obtenu le consentement libre et éclairé de ceux-ci. La participation à cette étude était volontaire sans aucune compensation financière et pouvait être interrompu à n'importe quel moment. L'inconfort majeur de cette étude était le temps accordé la réponse aux questions qui n'excédait pas 15 mn.

## Résultats

**Caractéristiques socio-professionnelles:** 120/245 praticiens ont répondu soit un taux de réponse global de 49% (83% pour les obstétriciens versus 37% pour les sages-femmes). Les répondants étaient au nombre de 120 dont 66 sages-femmes et 54 obstétriciens. Plus de la moitié des obstétriciens (55,6%) avaient moins de 5 années d´expérience, tandis que 51,5% des sages-femmes avaient entre 5 et 10 années d´expérience. Un pourcentage de 79,6% des obstétriciens et 37,9% des sages-femmes officiaient dans un centre hospitalier universitaire (Tableau 1).

**Tableau 1:** niveau de connaissances des 120 prestataires de soins

Items de connaissance connus	Sages-femmes		Obstétriciens	
	n	%	n	%
**Connaissance des facteurs de risques périnéo-sphinctériens**				
Parité	65	98,5	54	100,0
Gestité	60	90,9	46	85,2
Épisiotomie médiane	42	63,6	48	88,9
Prise de poids pendant la grossesse	20	30,3	34	63,0
Expression abdominale	20	30,3	45	83,3
Macrosomie fœtale	19	28,8	28	53,7
Présentation postérieure	18	27,3	32	59,3
Extraction instrumentale	13	19,7	50	92,6
Effort expulsif sur une vessie pleine	10	15,1	19	35,2
Durée de la 2^e^ phase du travail	8	12,1	34	63,0
Césarienne après dilatation > 8 cm	8	12,1	52	96,3
Obésité	3	4,6	32	59,3
Sport de haut niveau	2	3,0	11	20,4
**Connaissance sur la rééducation périnéale**				
Rééducation périnéale lutte contre les troubles du plancher pelvien	20	30,3	52	96,3
Rééducation périnéale utile en obstétrique	13	19,7	50	92,6
Connaissance de la rééducation périnéale	8	12,1	34	63,0
Rééducation périnéale facilite l´accouchement	10	15,1	34	63,0
**Moyens de rééducation périnéale connus**				
Gymnastique hypopressive	2	3,0	32	59,3
Biofeedback périnéal	2	3,0	23	42,6
Électrostimulation	-	-	11	20,4
Exercices de Kegel	1	1,5	10	18,5
Cône et sphères vaginaux	1	1,5	9	11,1
Eutonie	2	3,0	9	11,1
Méthode CMP*	1	1,5	2	3,7

* Connaissance et maitrise du périnée

**Connaissances:** la parité, l´extraction instrumentale, l´expression abdominale étaient les facteurs de risques périnéo-sphinctériens les plus cités des sages-femmes et obstétriciens. Une sage-femme sur dix (12%) avait une connaissance sur la rééducation périnéale du post partum. La gymnastique hypopepsie abdominale (59%) et le biofeedback (26%) étaient les plus connus des obstétriciens. Au total, l´indice de connaissance des sages-femmes était bon ou moyen dans 25% (n=14) des cas (IIQ 16,8-48,3). Les obstétriciens avaient un niveau de connaissances bon ou moyen dans 65% (n=43) des cas (IIQ de 41,1-48,3) (Tableau 2).

**Tableau 2:** indice de connaissance des 120 prestataires de soins

Indice de connaissance	Sages-femmes		Obstétriciens	
	N	%	n	%
Mauvais	23	41,8	7	10,6
Insuffisant	18	32,7	16	24,2
Moyen	11	20,0	31	47,0
Bon	3	5,4	12	18,2
Total	55	100,0	66	100,0

**Attitudes pratiques:** la majorité des prestataires convoquaient les femmes dans le post partum, mais seulement 7 soignants sur dix contrôlaient le périnée et deux obstétriciens sur dix proposaient une rééducation périnéale (Tableau 3).

**Tableau 3:** attitudes pratiques des 120 prestataires de soins

Items de pratique	Sages-femmes		Obstétriciens	
	n	%	n	%
**Recherche de l'incontinence urinaire comme signe fonctionnel pendant la grossesse**				
Jamais	42	63,6	2	3,7
Quelques fois	15	22,7	19	35,2
Souvent	9	13,6	16	29,6
Constamment	-	-	17	31,5
**Éducation des patientes sur l'incontinence urinaire**	65	98,5	45	83,3
**Proposition de la rééducation périnéale pendant la grossesse**	-	-	13	28,9
**Convocation des patientes dans le post-partum**				
Jamais	1	1,5	-	-
Quelques fois	4	6,1	-	-
Souvent	10	15,1	6	11,1
Constamment	51	77,3	48	88,9
**Motifs de convocation des patientes dans le post-partum**				
Évaluations des complications dans le perpartum	16	24,2	49	90,7
Soins de manœuvres obstétricales	2	3,0	4	7,4
Contrôle du périnée	47	71,2	38	70,4
**Délai des RDV**				
<4 semaines	19	28,8	9	16,7
4-8 semaines	43	65,1	45	83,3
>8 semaines	3	4,6	-	-
**Consultation spontanée dans le post partum**				
Jamais	6	9,1	3	5,6
Quelques fois	9	13,6	11	20,4
Souvent	44	66,7	21	38,9
Constamment	7	10,6	19	35,2
**Motifs consultations spontanée**				
Gratitude	43	65,1	32	59,3
Plaintes morphologiques	1	1,5	1	1,8
Plaintes périnées	6	9,1	11	20,4
Douleurs abdominales	4	6,1	2	3,7
**Observance des rendez-vous de consultation**				
Bonne	46	69,7	42	77,8
Mauvaise	19	28,8	13	22,2
**Proposition de la rééducation périnéale dans le postpartum**	-	-	11	20,4
**Méthodes de rééducation périnéale proposées**				
Exercices de Kegel	-	-	6	11,1
Gymnastique hypopressive	-	-	3	5,6
Biofeedback périnéal	-	-	-	-
Électrostimulation	-	-	-	-
Cône et sphères vaginaux	-	-	4	7,4
Méthode CMP*	-	-	-	-
Eutonie	-	-	-	-

* Connaissance et maitrise du périnée

## Discussion

L´intérêt de la rééducation périnéale était connu de 96,3% (n=52) des obstétriciens et 30,3% (n=20) des sages-femmes. Selon Deffieux *et al*. [12], chez les femmes qui ont une incontinence urinaire persistante à 3 mois du post-partum, une rééducation périnéale par des exercices de contraction volontaire des muscles du plancher pelvien est recommandée, sans qu´il soit possible d´en préciser les modalités précises. Des exercices de contraction volontaire des muscles du plancher pelvien sont à réaliser par la patiente elle-même. Une supervision est recommandée avec au minimum 3 séances avec un thérapeute (kinésithérapeute ou sage-femme), associées à des exercices réalisés au domicile [12].

La gymnastique hypopressive était le moyen de rééducation périnéale le plus connu de nos enquêtés. La gymnastique abdominale hypopressive est le fruit de recherches cliniques expérimentales et fondamentales en neurophysiologie initiées il y a plus de 25 ans par Marcel Caufriez. Si les techniques hypopressives ont indiscutablement une place dans l´arsenal thérapeutique de la rééducation périnéale de l´incontinence urinaire d´effort, son rôle est prépondérant en aval: la prévention. Inclus dans le grand ensemble de la rééducation neuromyostatique, « la gymnastique hypopressive est un ensemble agencé d´exercices posturaux rythmés qui permet l´intégration (centre vestibaux cérébelleux) et la mémorisation (cortex somesthésie) des messages proprioceptifs sensitifs et sensoriels associés à une situation posturale particulière». Sa pratique provoque une chute de la pression intra-abdominale dont le rôle est capital pour son activation réflexe (de type I) du plancher pelvien et de la sangle abdominale.

En conséquence sa pratique quotidienne à long terme répond d´une manière parfaite aux problématiques du post partum tout en ayant l´avantage de normaliser des tensions des muscles anti-gravitaires et pariétaux impliqués dans l´équilibre postural corporel. Le biodfeedback était le deuxième moyen de rééducation le plus connu de nos enquêtés. Le principe est la prise de conscience précise de la contraction périnéale par l´intermédiaire de signaux le plus souvent visuels et parfois auditifs à l´aide d´une sonde adaptée à ce type de rééducation et qui doit être placée au niveau vaginal [13].

Environ deux tiers des sages-femmes (63,6%) (n=42) ne recherchaient jamais l´incontinence urinaire comme signe fonctionnel pendant la grossesse. Par ailleurs, elles ne proposaient jamais de rééducation périnéale aux patientes. Cette méconnaissance de pratique pourrait justifier le taux de non-réponses élevé chez les sages-femmes (63%) malgré les multiples relances par appel téléphonique et mails. En ce qui concerne les obstétriciens, 31,5% (n=17) d´entre eux recherchaient constamment l´incontinence urinaire comme signe fonctionnel pendant la grossesse. Les méthodes de rééducation périnéale proposées étaient l´exercice de Kegel (11,1%) et la gymnastique hypopressive (5,6%). Le biofeedback n´était pas mentionné par les praticiens comme méthode de rééducation périnéale volontairement ou non. En effet, dans notre pays, une seule structure publique logée dans l´un des CHU de la ville propose cette offre de soins. Alors que, la plupart de nos répondants officiaient dans des structures publiques, ceci pouvant justifier notre observation. Tandis que certains d´entre eux connaissaient cette méthode mais ne la proposaient pas. Cette attitude serait probablement due aux niveaux de preuve insuffisante pour fournir des recommandations solides sur la meilleure façon d'aborder les TPP [14-16], en particulier, sur les résultats à long terme font défaut [17-19]. Une enquête européenne récente auprès de 26 pays a révélé que seulement un tiers des urogynécologues utilisaient le biofeed back dans le traitement des incontinences urinaires, malgré le fait que 92% considéraient que le biofeedback était une valeur ajoutée [20]. Ainsi, cette offre de soins qui nécessite des suppléments financiers est volontairement délaissée compte tenu du contexte socio-économique des patientes.

Cette étude connait des limites à cause de l´hétérogénéité des sujets enquêtés sur le plan sociodémographique et professionnel. Aussi, les habitudes des praticiens varient selon leur formation et le plateau technique disponible sans oublier la diversité socio-économique et culturelle de la population cible. Il était donc impossible de réaliser des analyses uni ou multivariées par catégorie. Autre facteur limitant, le faible taux de répondants, malgré la simplification des questions et la réduction de celles-ci à 21. Comme tout sondage d´opinion, le taux de répondants reste un défi. Ainsi, malgré toute la logistique informatique et les multiples rappels, le taux de réponses lors d´une enquête européenne était de 18 à 30% [20]. Par contre, leur questionnaire comprenait 51 items. Tout ceci traduit la complexité de la mise en œuvre d´une telle étude. Malgré tout, notre étude peut être considérée comme un travail préliminaire jetant les bases d´un travail à grande échelle avec une meilleure méthodologie.

## Conclusion

Le faible niveau de connaissances des sages-femmes et obstétriciens ivoiriens sur la rééducation périnéale du post partum et le manque de pratique suggèrent la nécessité d´une mise à niveau perpétuelle de ceux-ci à travers des formations continues. D´autant plus que des options de traitements innovantes adaptés à notre contexte sont en cours d´étude.

### Etat des connaissances sur le sujet

- La grossesse et l'accouchement sont les principaux facteurs étiologiques des troubles sphinctériens;
- La rééducation périnéale du post partum est un moyen thérapeutique utilisé dans le cadre de la prise en charge des troubles sphinctériens.

### Contribution de notre étude à la connaissance

- Le niveau de connaissance des obstétriciens ivoiriens sur la question de la rééducation périnéale du post partum était moyen et faible en ce qui concerne les sages-femmes malgré un calcul d´un indice de connaissance adapté à notre contexte africain;
- La promotion de la recherche africaine sur l´intérêt de la rééducation périnéale dans la prise en charge des troubles sphinctériens.

## Remerciements

Les auteurs remercient la Société de Gynécologie Obstétrique de Côte d´ivoire (SOGOCI) qui a favorisé l´identification des prestataires de soins et la distribution des questionnaires.
